# M1-like tumor-associated macrophages activated by exosome-transferred THBS1 promote malignant migration in oral squamous cell carcinoma

**DOI:** 10.1186/s13046-018-0815-2

**Published:** 2018-07-09

**Authors:** Meng Xiao, Jianjun Zhang, Wanjun Chen, Wantao Chen

**Affiliations:** 1 Department of Oral and Maxillofacial-Head and Neck Oncology, Shanghai Ninth People’s Hospital, Shanghai Jiao Tong University School of Medicine, 639, Zhizaoju Road, Shanghai, 200011 China; 2 Shanghai Research Institute of Stomatology and Shanghai Key Laboratory of Stomatology, Shanghai, 200011 China; 3 Mucosal Immunology Section, NIDCR, NIH, Bethesda, MD 20892 USA

**Keywords:** Macrophage, Exosome, THBS1, Oral squamous cell carcinoma, Migration

## Abstract

**Background:**

Treatment strategies targeting tumor-associated macrophages (TAMs) have been proposed in cancer areas. The functional alterations of macrophages in the microenvironment during the tumorigenesis of human epithelial cancer remain poorly understood. Here, we explored phenotypic alteration of macrophages during the development of oral squamous cell carcinoma (OSCC).

**Methods:**

Conditioned media (CM) and exosome supernatants were harvested from normal oral epithelium, oral leukoplakia cells and OSCC cells. We measured phenotypic alteration of macrophages using flow cytometry, luminex assays, and quantitative real-time PCR assay. Intracellular signaling pathway analysis, mass spectrometry proteomics, western blotting, enzyme-linked immunosorbent assay, immunohistochemical staining, and bioinformatics analysis were performed to uncover the underlying mechanisms.

**Results:**

THP-1-derived and PBMCs derived macrophages exhibited an M1-like phenotype but not M2-like phenotype, when treated with CM from OSCC cells but not with the CM from normal epithelium or leukoplakia cells. Further investigations revealed that macrophages were activated by taking up exosomes released from OSCC cells through p38, Akt, and SAPK/JNK signaling at the early phase. We further provided evidences that THBS1 derived from OSCC exosomes participated in the polarization of macrophages to an M1-like phenotype. Reciprocally, CM from exosomes induced M1-like TAMs and significantly promoted migration of OSCC cells.

**Conclusions:**

We proposed a novel paracrine loop between cancer cells and macrophages based on exosomes from OSCC. Therefore, target management of M1-like TAMs polarized by exosomes shows great potential as a therapeutic target for the control of cancerous migration in OSCC.

**Electronic supplementary material:**

The online version of this article (10.1186/s13046-018-0815-2) contains supplementary material, which is available to authorized users.

## Background

The immune system is an indispensable regulator in the crosstalk between cancer cells and tumor microenvironment [1, 2]. Among immunological effector cells associated with tumor microenvironment, macrophages have been widely recognized to participate in cancer-related inflammation, immune escape, matrix remodeling, and cancer metastases [3–5]. Over the years, it has been reported that macrophages account for 5–40% of malignant solid tumors [6, 7]. Macrophages display considerable functional plasticity in response to local microenvironment stimuli [8]. Activated macrophages are functionally classified into two populations in vitro, M1 and M2 [9–11]. Tumor-associated macrophages (TAMs) are termed as a macrophage population recruited and educated by cancer cells, which exert important roles in tumor microenvironment [4, 12, 13]. Due to these findings, strategies targeting macrophages have been proposed in cancer therapy [14].

Canonically, TAMs are characterized by a molecular signature consistent with that of M2 macrophages [6, 15, 16]. Recently, increasing evidence suggests that TAMs are not composed of a homogeneous population but are a mixed population of macrophages harboring both M1 and M2 phenotypes that have been detected in several malignant solid tumors [17–19]. In hepatocellular carcinoma, CD68(+) HLA-DR(+) M1-like TAMs were shown to suppress anti-tumor immunity and promote cancer metastasis through expression of B7-H1 [17]. In pancreatic ductal adenocarcinoma, TAMs were reported to exhibit M1 and M2 properties, both of which promoted the epithelial-mesenchymal transition [20]. In addition, a mixed population of macrophages with M1 and M2 phenotypes was detected in vitro in several types of cancer cells [12, 18, 19]. However, no study has elucidated the underlying mechanism of these alterations for M1- or M2-like TAMs, especially with respect to the M1-like polarization. Thus, it is urgent to fully understand the education of TAMs when considering the heterogeneity among various tissue-derived cancers.

Oral squamous cell carcinoma (OSCC), belonging to the head and neck squamous cell carcinoma (HNSCC), remains one of the most lethal cancers worldwide, involving the mucosa epithelial cells from the oral cavity [21]. Development of OSCC is evolutionary and characterized by specific transformation from normal epithelium to epithelial precancerous condition, and to cancerous lesion [22]. In malignant solid tumors, emerging evidence supports the notion that many secretory products from cancer cells participate in the education and polarization of macrophages through paracrine loops [3, 23]. However, the functional alterations of macrophages during the developing of OSCC are poorly understood. Herein, we aimed to investigate the phenotypic alterations of macrophages in the tumor microenvironment during the developing of OSCC.

## Methods

### Cell line cultures and cancer-conditioned media preparation

Human immortal oral epithelial cell line cells (HIOEC) and oral leukoplakia cell line cells (Leuk1) were cultured in keratinocyte serum-free media [24, 25]. OSCC cell lines SCC25 and Cal27 were obtained from American Type Culture Collection and cultured in DMEM supplemented with 10% FBS, penicillin (100 U/ml) and streptomycin (100 μM/ml) at 37 °C in the presence of 5% carbon dioxide [26]. To obtain conditioned media (CM) of various cell lines, cultured cells, up to 80% confluence, were washed and cultured for an additional 24 h with fresh RPMI 1640 media [19]. The cell-free supernatants were collected and centrifuged at 3900 rpm (4 °C) for 15 min. The CM was finally harvested after successively filtration through 0.45 μm and 0.22 μm Filter Units.

Stable THBS1 knockdown cells for SCC25 and Cal25 were generated by transfection with THBS1-specific short hairpin RNA (5’-TTC TCC GAA CGT GTC ACG T -3′ for Scrambled, 5′-GTA GGT TAT GAT GAG TTT AAT -3′ for sh1, 5′-GGA CAA CTG TCC ATT CCA TTA -3′ for sh2) lentivirus, and positively selected with puromycin (10 μg/mL, Calbiochem, USA).

### Macrophage differentiation and polarization

THP-1 cells were obtained from ATCC and maintained in RPMI 1640 media supplemented with 10% FBS, penicillin (100 U/ml) and streptomycin (100 μM), and 2-mercaptoethanol (2-ME, 50 μM, Sigma-Aldrich, USA). To obtain resting macrophages (M0), THP-1 cells were differentiated under phorbol-12-myristate-13-acetate (PMA, 100 ng/ml, Sigma-Aldrich, USA) treatment for 24 h and rested for another 24 h [19, 27]. The M0 cells were polarized into M1 using 50 ng/ml IFN-γ (PeproTech, USA) and 1 μg/ml LPS (Sigma-Aldrich, USA) for 24 h, and polarized into M2 using IL-4 and IL13 (20 ng/ml, PeproTech, USA) for 7 days [28, 29].

Peripheral blood mononuclear cells (PBMCs) were obtained from healthy controls using a Ficoll-Hypaque (GE Healthcare, USA) density gradient [30]. Monocytes were isolated from PBMCs with Human CD14 MicroBeads (MiltenyiBiotec, USA) and positively selected by magnetic activated cell sorting (MACS) separation technique according to the manufacturer’s protocol. Purified monocytes were incubated for 7 days in RPMI 1640 medium supplemented with 10% FBS and 100 ng/mL of M-CSF to obtain M0 [31, 32].

### Exosome isolation and identification

The collected CM were filtered through 0.10 μm Filter Units, then transferred to Amicon® Ultra-15 centrifugal filter device (100 K, Merck Millipore, USA) and concentrated by centrifugation at 3900 rpm at 4 °C for 15 min. Exosomes were then carefully washed with PBS, and resuspended in PBS or RPMI 1640 fresh medium for validation or subsequent experiments [33]. Freshly isolated exosomes were diluted 1:1000 for nanoparticle tracking analysis (NTA) measurements (NanoSight NS500, NTA 3.2 Dev Build 3.2.16). Size distribution and quantification of exosome samples were analyzed with NanoSight LM10 system (NanoSight, Wiltshire, UK). Size distribution of the detected exosomes was determined and was represented as mean ± SD [34].

### Macrophage morphology and imaging

Morphologies of treated macrophages were observed and photographed under an inverted microscope (ZEISS, German). For fluorescent observation, macrophages were fixed in paraformaldehyde and permeabilized beforehand. The cytoskeleton was labeled with Acti-stain™ 555 Fluorescent Phalloidin (Cytoskeleton, USA) and nuclei with DAPI (YEASEN, China). Fluorescently labelled cells were examined using a ZEISS fluorescent imaging microscope (ZEISS, German).

### Fluorescent labelling of exosomes and tracing exosome uptake by macrophages

Isolated exosomes were incubated with Exo-Red labelling reagent (Exo-Glow Exosome Labeling Kits, System Biosciences, USA) for 30 min at 37 °C. Labeled exosomes were washed with PBS using an ultra-filter device, and suspended in fresh RPMI 1640 media. Exosomes labeled with Exo-Red dyes were added to THP-1 derived or PBMCs derived M0 cells and incubated overnight. Subsequently, cells were washed out to remove free exosomes, and then fixed, permeabilized, and stained with Acti-stain™ 488-Phalloidin and DAPI as described above. Exosome uptake by macrophages was examined using Nikon’s A1 confocal laser microscope (Japan).

### Flow cytometry

After treatment with CM or exosomes for 24 h, macrophages were washed, trypsinized, and resuspended in PBS containing 1% FBS. Next, cells were incubated with surface markers (FITC Mouse anti-Human CD14, Alexa Fluor Mouse anti-Human CD163, PE Mouse anti-Human CD86; FITC Mouse IgG2ακ, Alexa Fluor Mouse IgG1κ and PE Mouse IgG1κ used for Isotype Control; all from BD Biosciences, USA). For cell cycle analysis, cells were harvested and fixed in 70% ethanol at 4 °C and stained with propidium iodide (PI, BD Biosciences, USA). After staining, cells were analyzed by flow cytometry (Beckman CytoFLEX FCM, USA).

### Enzyme-linked immunosorbent assay (ELISA)

Cytokine analyses were performed with Luminex™xMAP technology using High Sensitivity 9-Plex Human ProcartaPlex™ Panel (ThermoFisher Scientific, USA). Culture supernatants from treated macrophages were harvested, filtered, and stored at − 80 °C prior to analysis according to the protocols provided by the manufacturer. All samples were run in triplicate. THBS1 levels were quantified in cell culture supernatants and exosome suspensions using a Platinum ELISA assay according to the manufacturer’s instructions (eBioscience, USA).

### Quantitative real-time PCR (qRT-PCR) assay

Total RNA was extracted and reversely transcribed using the PrimeScript RT reagent Kit (TaKaRa, Japan) according to the protocols recommended by the manufacturer. The cDNA was subjected to qRT-PCR detection using a SYBR Green Premix Kit (TaKaRa, Japan). The relative expression was calculated using the 2^-ΔΔCT^ method for the following genes: TNFα, IL1β, IL6, IL10, CCL18, MRC1, CD80, HLA-DRα, PAI1, and THBS1.

### Intracellular signaling pathway analysis

THP-1-derived macrophages were lysed after treatment with exosomes for 24 h. Cell lysates were assayed using the PathScan Immune Cell Signaling Antibody Array Kit according to the manufacturer’s protocol (Cell Signaling Technology, USA). Subsequently, THP-1-derived macrophages were lysed after treatment with exosomes for 2 h and 6 h, and cell lysates were assayed using the PathScan Intracellular Cell Signaling Antibody Array Kit (Cell Signaling Technology, USA) accordingly.

### Mass spectrometry (MS)-based label-free quantitative proteomics

Exosomes were harvested and lysed for mass spectrometry-based label-free quantitative proteomics analysis by Beijing BangFei Bioscience Co., Ltd. [35]. Eluted peptides from each sample underwent data acquisition in an Orbitrap Fusion mass spectrometer (Thermo Scientific, USA). MS RAW data files were uploaded into the Mascot 2.1 via Proteome Discoverer, and the referred database was uniprot-human_160701.fasta. Functions of the filtered proteins were analyzed through the UniProtKB database.

### Western blotting

For immunoblotting, cellular extracts or exosomes extracts were acquired by using RIPA Lysis Buffer containing proteinase inhibitor cocktail (Innovation, USA). After subjecting the lysates to SDS-PAGE electrophoresis, proteins were transferred onto a polyvinylidene difluoride membrane by electroblotting. The membranes were then blocked and incubated with primary antibodies (anti-Alix antibody, from Thermo Fisher; anti-CD9 and anti-CD63 antibodies from System Biosciences, USA; anti-Rab5 antibody, from BioVision; anti-phospho-Akt (Ser473), anti-Akt (pan), anti-phosphor-SAPK/JNK (Thr183/Tyr185), anti-SAPK/JNK, anti-phosphor-p38 MAPK (Thr180/Tyr182), and anti-p38 MAPK antibodies, from Cell Signaling Technology, USA; anti-β-tubulin antibody, from BOSTER Biological Technology, China). Specific antibody-bound protein bands were detected with ECL Plus reagent (Millipore, USA) under Amersham Imager 600 (GE, USA).

### Immunohistochemical staining

Sections of 5 μm were prepared from paraffin-embedded samples. After deparaffinization, rehydration, and antigen retrieval, endogenous peroxidase activity was quenched. Immunohistochemistry (IHC) staining was performed with primary antibody (mouse anti-human THBS1 antibody and mouse anti-human CD68 antibody from Santa Cruz, USA; rabbit anti-human CD80 antibody from Abcam, USA). For THBS1 staining, the samples were incubated with a biotinylated secondary antibody followed by staining with a DAB kit (GTVision, China). For CD68 and CD80 double-staining, a multiplex mouse-HRP/rabbit-AP IHC kit (Enzo Life Sciences, Switzerland) was used according to the protocol provided by the manufacturer. The proportion of CD68^+^ CD80^+^ areas in CD68+ areas were measured in 20 OSCC cases by using the freeware Image J Version 1.51 t, downloaded from the National Institutes of Health (NIH) website (https://imagej.nih.gov/ij/).

### Bioinformatics analysis and validation

Bioinformatics analysis was performed based on the TCGA HNSCC cohort using the UCSC Xena Browser [36, 37]. In total, 604 cases were searched for gene expression under RNAseq (polyA+IlluminaHiSeq). Only cases of primary HNSCC were filtered and included for further analysis of expression patterns of THBS1, TNFα, IL6, IL1B, CD68, CD80, and CD86. Expression heat-maps of defined gene sets were generated and clustered online, and detailed data were downloaded for subsequent statistical analysis. A validated cohort was constructed based on 30 primary OSCC cases. The patients involved in this study signed written informed consent, and the study was approved by the Medical Ethics Committee of the Ninth People’s Hospital, Shanghai Jiao Tong University School of Medicine.

### Cell migration assays

Transwell assays were performed to examine the migration ability of OSCC cells in response to the CM of exosome-treated macrophages. Cancer cells were suspended in 200 μl (5 × 10^4^ cells) of fresh medium and plated into Millicell chambers (8 μm, Millipore Corporation, USA) with 300 μl of culture media containing 10% FBS and 300 μl CM from exosome-treated macrophages in the bottom chamber. After 24 h, cells that migrated through the filter were fixed with paraformaldehyde, stained with 10% crystal violet. The migrated cells were photographed and counted.

### Statistical analysis

All statistical analyses in this study were conducted with SPSS16.0 software (SPSS, Inc., Chicago, IL, USA), and data are presented as the mean ± SD. The significant difference between two groups was determined by Student’s *t*-test. Pearson Chi-squared tests were performed to assess the statistical significance for correlations between two variables. A *p*-value < 0.05 was considered statistically significant.

## Results

### Conditioned media from OSCC cells polarizes macrophages to the M1-like phenotype

**Fig. 1:**
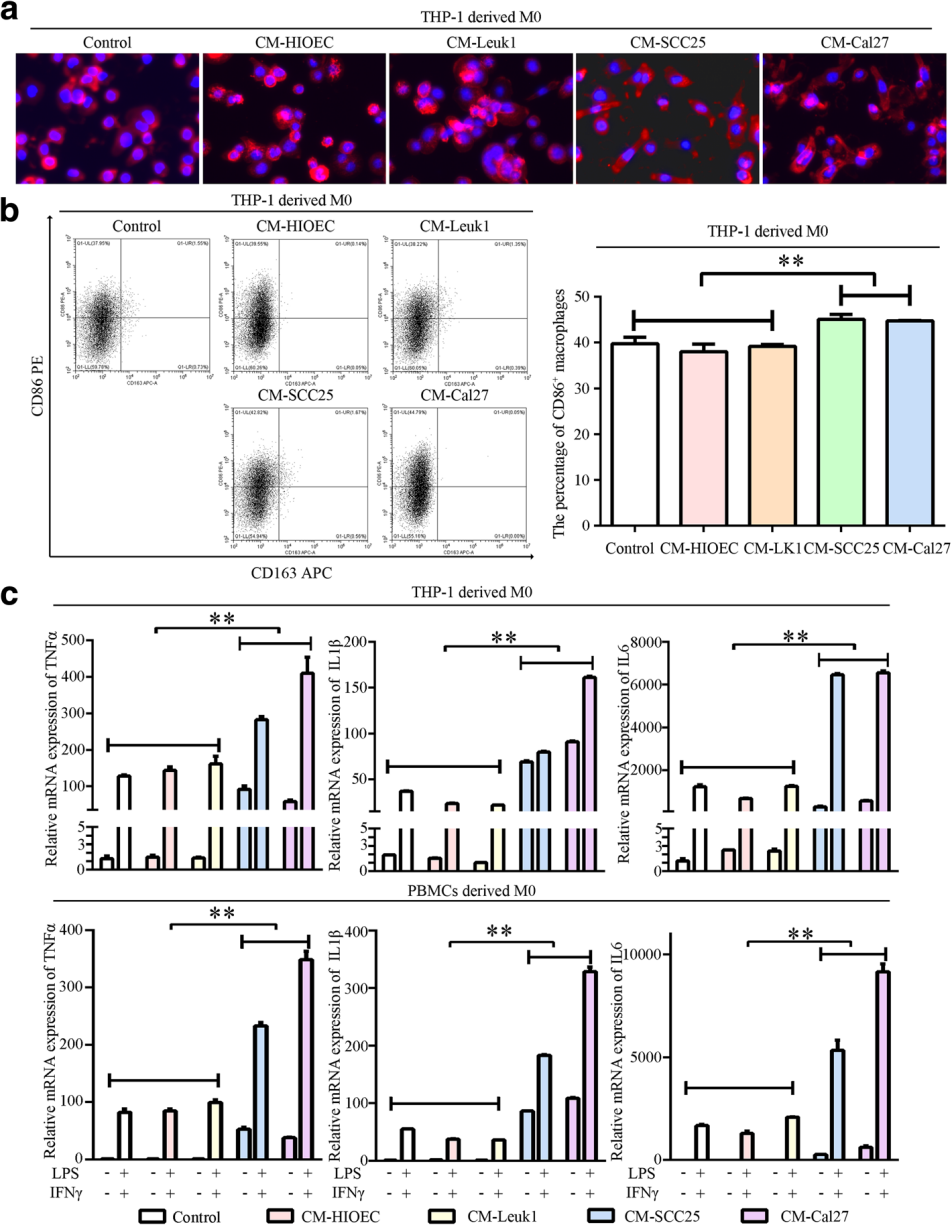
Characterization of macrophages educated by CM. **a**. Representative morphological images of THP-1-derived macrophages educated by CM from HIOEC, Leuk1, SCC25, and Cal27 for 24 h. The cytoskeleton was labeled with Fluorescent 555-Phalloidin (Red), and Nuclei were labeled with DAPI (Blue). **b**. Representative Scatter plots showed CD86 and CD163 levels evaluated by flow cytometry in THP-1-derived macrophages educated by CM from HIOEC, Leuk1, SCC25, and Cal27 cells for 24 h. The histogram showed statistical analysis for percentages of CD86^+^ macrophages. **c**. Expression levels of TNFα, IL1β, and IL6 of THP-1 derived and PBMCs derived macrophages educated by CM from HIOEC, Leuk1, SCC25, and Cal27 for 24 h, with or without subsequent LPS and IFNγ stimuli for another 24 h, as determined by quantitative real-time PCR. Data were represented as the mean ± SD of three independent experiments, **p < 0.01

The development of OSCC from normal epithelium is characterized by a specific transformation process at the precancerous condition. To investigate functional and phenotypical plasticity of macrophages during the transformation of OSCC, we performed our studies by using HIOEC, Leuk1, SCC25 and Cal27. THP-1-derived macrophages appeared round and smooth in response to incubation with CM from HIOEC and Leuk1, similar to M0 macrophages, whereas the macrophages were rough and branched when incubated with CM from SCC25 and Cal27 (Fig. 1a). By detecting the expression of M1 marker CD86 and M2 marker CD163, we observed a significant increase in the frequency of CD86^+^ cells in the macrophages pre-treated with CM from SCC25 and Cal27 compared to CM from HIOEC and Leuk1 (Fig. 1b), indicating that the THP-1-derived macrophages exhibited an M1-like phenotype when treated with CM from OSCC cells but not CM from normal epithelium or leukoplakia cells. In addition, increased expression levels of representative M1 cytokines TNFα, IL1β, and IL6 were found in CM-SCC25 and CM-Cal27 treated THP-1-derived and PBMCs derived M0 (Fig. 1c). Moreover, treatment with CM-SCC25 and CM-Cal27 significantly enhanced the response of macrophages to LPS and IFNγ stimuli (Fig. 1c).

### Exosomes released from OSCC cells activate macrophages

**Fig. 2:**
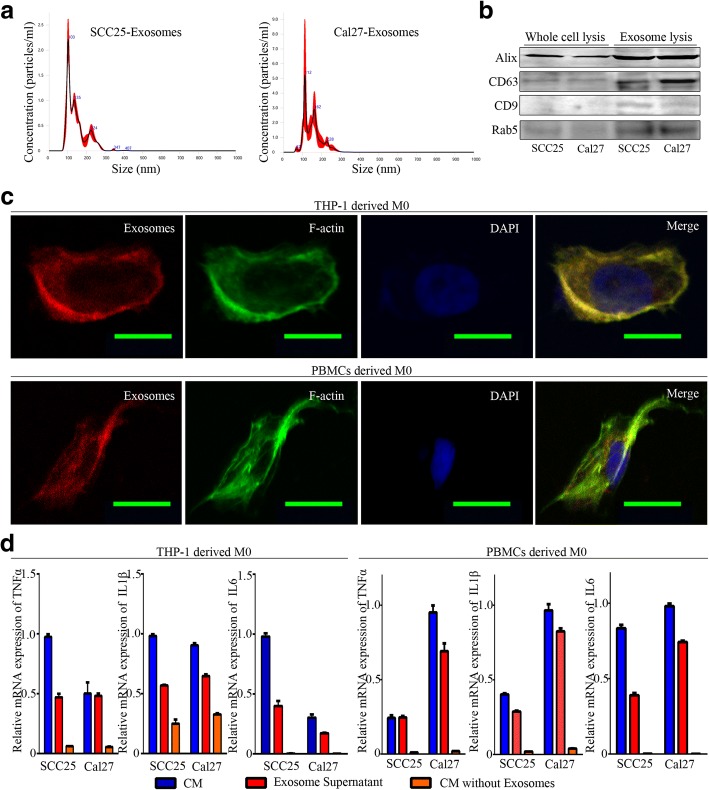
Exosome isolation from CM of OSCC cell lines. **a**. Freshly isolated exosomes were diluted 1:1000 for nanoparticle tracking analysis using Nanosight technology. The curve of the graph illustrated that the majority of exosomes were distributed with a peak at size 103 nm for SCC25-Exosoems and 112 nm for Cal27-Exosomes. Three independent experiments were performed, and results were merged into one curve. **b**. Bands represented the immunoblotting analysis of human Alix, CD63, CD9, and Rab5 on whole cell lysates and exosome lysates isolated from SCC25 and Cal27. **c**. Uptake of exosomes by THP-1-derived and PBMCs derived macrophages. Macrophages were labeled with 488-Phalloidin (green), exosomes with Exo-Red (Red), and Nuclei with DAPI (Blue), bar = 25 μm. Negative control was presented in Additional file 3. D. Transcription levels of TNFα, IL1β, and IL6 in THP-1-derived and PBMCs derived macrophages educated by conditioned media, exosome supernatant or conditioned media without exosomes from SCC25 and Cal27 for 24 h. Data were represented as the mean ± SD of three independent experiments.

Extracellular vesicles are another major component that exists in CM in addition to soluble factors (like cytokines, chemokines, and growth factors), and among such vesicles, exosomes have been recognized as key mediators for intercellular communication [38, 39]. To elucidate the functional effectors in CM, we managed to isolate and identify exosomes from the CM of OSCC cell lines (Fig. 2a, b). Previous studies have reported that macrophages are key players in exosomes fast uptake and information transfer [40, 41]. We visualized and traced the uptake of fluorescence-labeled exosomes using confocal fluorescence microscopy. We observed the uptake of OSCC-derived exosomes by THP-1-derived macrophages and PBMCs derived macrophages (Fig. 2c), suggesting that macrophages could accept the biological signals from exosomes of OSCC by direct uptake. We used CM, equal amount exosome supernatant, and CM without exosomes to stimulate macrophages, respectively. Comparatively, exosomes played the most roles in regulating the expression of M1 signature genes (TNFα, IL1β, and IL6) in both THP-1 derived and PBMCs derived macrophages, but not the exosome-free CM from SCC25 and Cal27 (Fig. 2d).

**Fig. 3:**
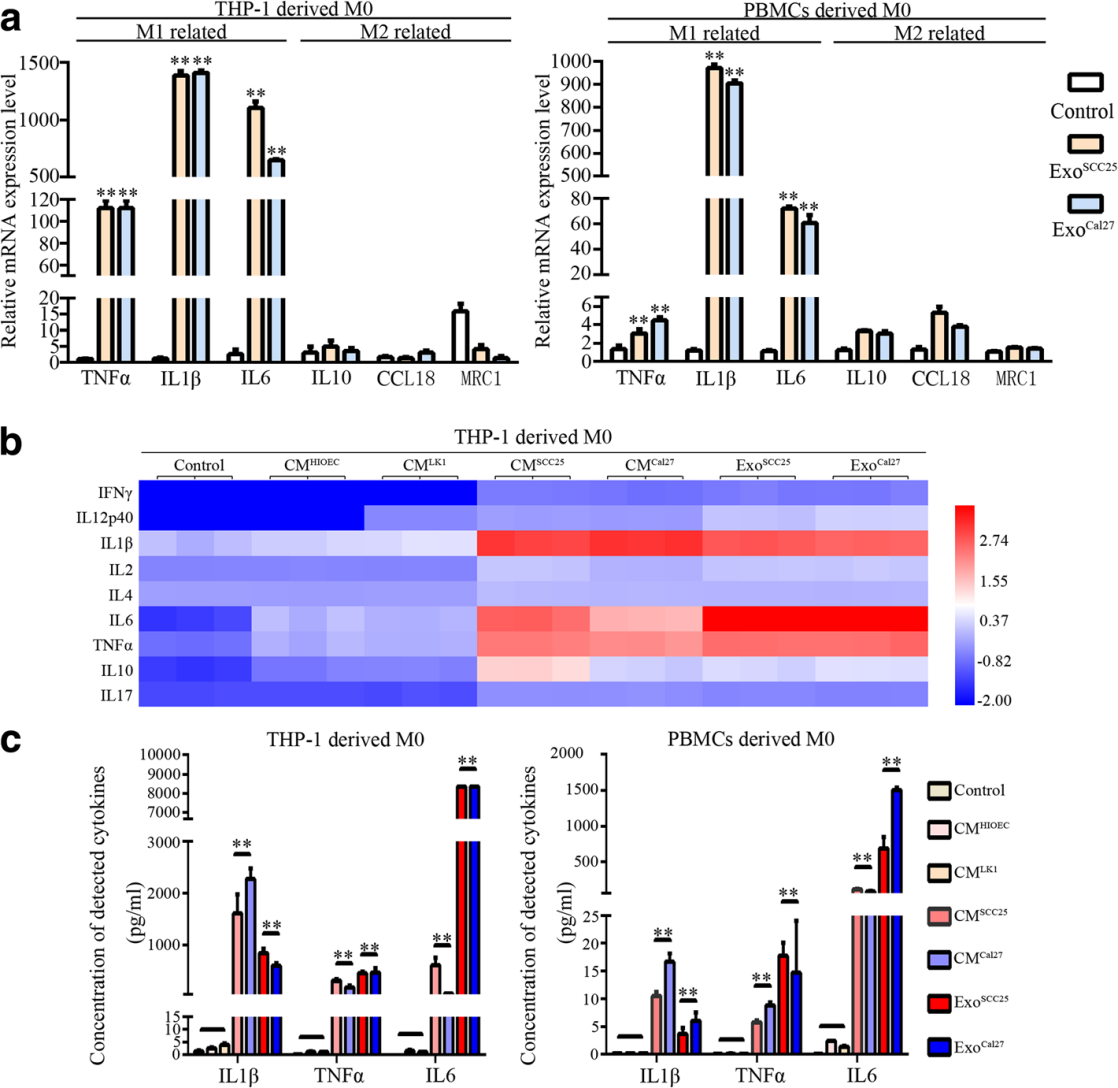
Exosomes from OSCC cell lines activated macrophages into the M1-like phenotype. **a**. Expression of representative M1 and M2 marker genes determined by qPCR in THP-1-derived and PBMC-derived macrophages after stimuli with exosome supernatants of SCC25 and Cal27 for 24 h. **b**. Heat-map of cytokine expression profiles assessed by Luminex assay. THP-1-derived macrophages were treated with CM from HIOEC, Leuk1, SCC25, and Cal27, and exosome supernatant from SCC25 and Cal27 cells for 24 h, respectively. All samples were run in triplicate. Colors illustrated fold changes (see color scale). Red: up-regulation; blue: down-regulation. **c**. Detection of IL1β, TNFα, and IL6 levels in culture supernatants of THP-1-derived and PBMC-derived macrophages under education of CM from HIOEC, Leuk1, SCC25, and Cal27 cells, and exosome supernatant from SCC25 and Cal27 for 24 h, respectively. Data were expressed as the mean ± SD from three experiments, **p < 0.01, a significant difference compared with control, CM-HIOEC and CM-Leuk1 treated groups

We examined transcriptional levels of M1 and M2 signature genes in macrophages derived from THP-1 and PBMCs stimulated by enriched exosome-supernatants from SCC25 and Cal27. The expression levels of M1 related genes (TNFα, IL1β, and IL6) were significantly increased in the macrophages, while the expression levels of M2 related genes (IL10, CCL18, and MRC1) were not changed (Fig. 3a). Protein levels of these cytokines were assessed by Luminex assays, and the results showed that OSCC-derived CM and exosome supernatants significantly enhanced the secretion levels of these M1 related cytokines (Fig. 3b, c). These data indicated that exosomes in OSCC-CM activated macrophages into M1-like phenotype.

**Fig. 4:**
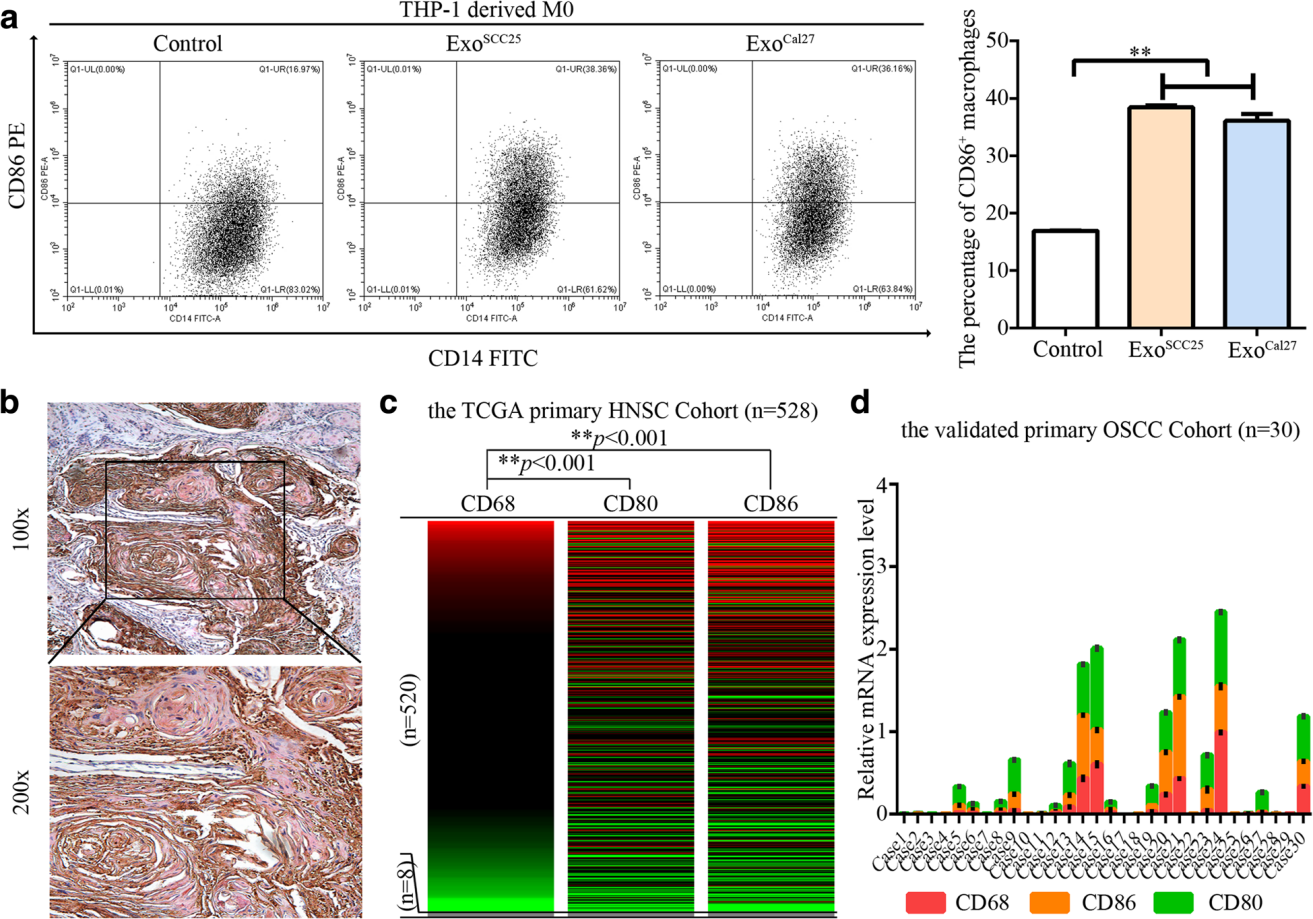
M1-like polarization in macrophages activated by OSCC exosomes and in OSCC samples.**a**. Representative Scatter plots showed CD86 expression levels in THP-1-derived macrophages stimulated by exosomes from SCC25 and Cal27 for 24 h determined by flow cytometry. The histogram showed quantification for the percentage of CD86^+^ macrophages. Data were presented as the mean ± SD of three independent experiments, **p < 0.01, a significant difference compared with the control. **b**. Chromogenic double staining with CD80 (pink)/CD68 (brown) in primary OSCC samples. Cells double positive for CD80 and CD68 were observed in OSCC samples. Representative images shown are in 100× and 200× magnification; control images were presented in Additional file 4. **c**. Heat-map from the UCSC Xena Browser based on the primary TCGA HNSCC cohort depicted the gene expression relationship between the CD68 pan surface marker for macrophages and the M1 polarization surface marker CD80 or CD86. **p < 0.01, a significant correlation with CD68 expression was observed for both CD80 and CD86.D. Expression pattern of CD68, CD86, and CD80 in a validated primary OSCC cohort (*n* = 30)

In addition, we observed that the percentage of CD86^+^ macrophages was significantly increased under the treatment of OSCC-derived exosome supernatants in vitro (Fig. 4a). In solid tissues, CD68 is used as pan-marker of macrophages, and co-expression of CD68 and CD80 indicates the M1-like macrophages [42]. Chromogenic double staining with CD80 and CD68 in primary OSCC samples revealed centered distribution of M1-like macrophages in CD68^+^ clusters among the cancer nests (Fig. 4b). Statistically, CD68^+^CD80^+^ areas accounted for about 31% (range: 22–45%) of all CD68^+^ areas in 20 samples of OSCC. Bioinformatics analysis based on the primary TCGA HNSCC cohort depicted significant co-expression between CD68 and CD80 or CD86 (Fig. 4c). In a validated cohort based on 30 primary OSCC cases, significant co-expression pattern between CD68 and CD80 or CD86 was also observed (*p* < 0.01, respectively, Fig. 4d). The above data demonstrated the extensive existence of M1-like macrophages in OSCC tissues.

### Exosomes from OSCC trigger M1-like macrophages through activation of p38, Akt, and SAPK/JNK signaling

**Fig. 5:**
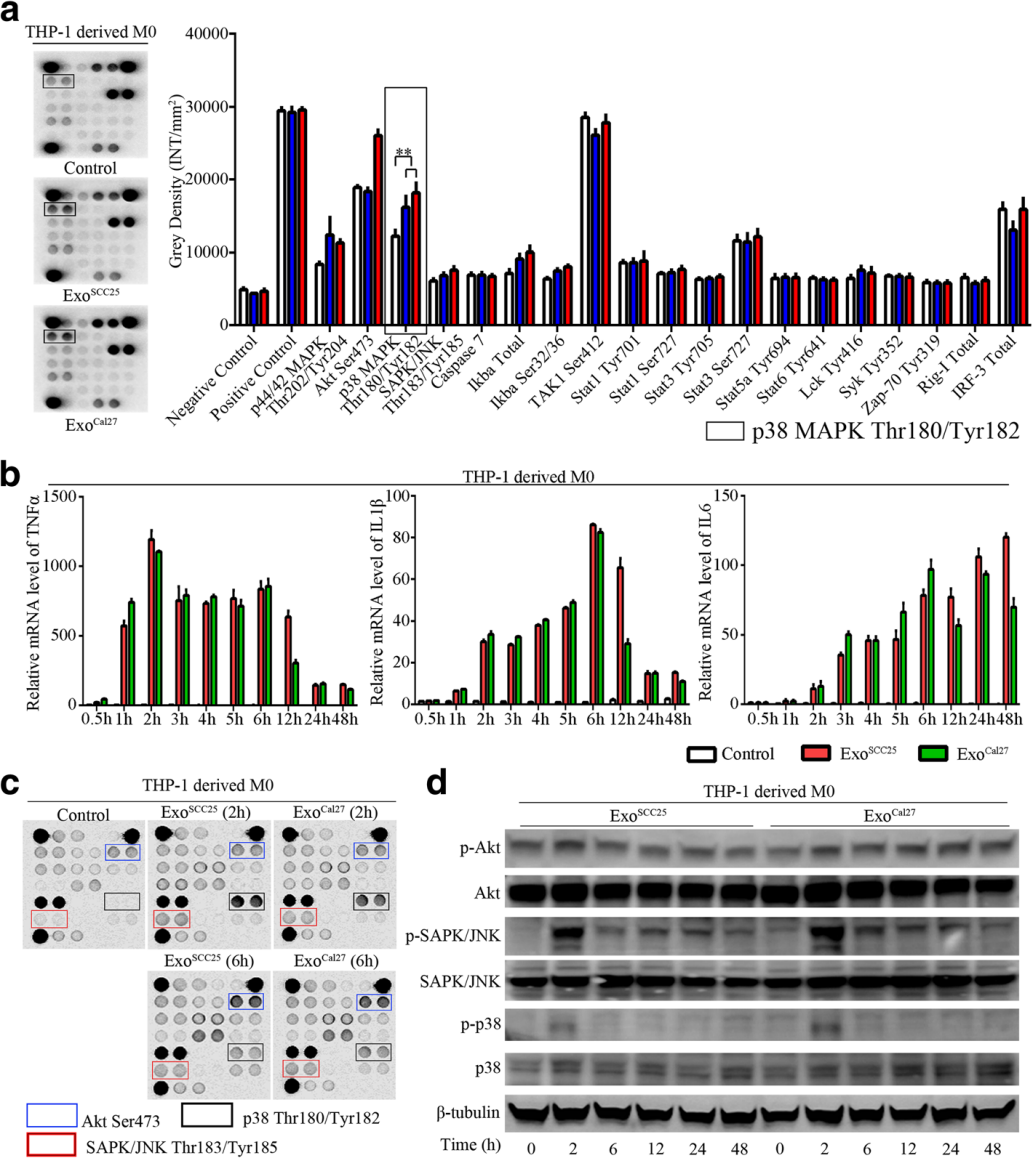
Cell signaling analysis for macrophages in response to exosome activation. **a**. THP-1-derived macrophages were lysed after treatment with exosomes for 24 h, and cell lysates were detected using the PathScan Immune Cell Signaling Antibody Array Kit by measuring densitometry values. Four independent experiments were performed, and representative images were shown. **p < 0.01, compared to control group. **b**. THP-1-derived macrophages were treated with OSCC exosomes for 0.5 h, 1 h, 2 h, 3 h, 4 h, 5 h, 6 h, 12 h, 24 h, and 48 h, respectively. The transcription levels of TNFα, IL1β, and IL6 were detected. Data are representative of three independent experiments. **c**. THP-1-derived macrophages were lysed after treatment with exosomes for 2 h and 6 h. Cell lysates were assayed using the PathScan Intracellular Cell Signaling Antibody Array Kit by measuring densitometry values. Activation of p38 MAPK Thr180/Tyr182 (black boxed area), Akt Ser473 (blue boxed area), and SAPK/JNK Thr183/Tyr185 (red boxed area) was observed. **d**. THP-1-derived macrophages were treated with OSCC-derived exosomes for 0 h, 2 h, 6 h, 12 h, 24 h, and 48 h. Cell lysates were harvested and analyzed by immunoblotting for the activation of Akt, SAPK/JNK, and p38 signals

Activation of potent intracellular signaling in response to various extracellular stimuli is essential for macrophage polarization. We next examined the underlying molecular mechanisms for the activation of macrophages treated by exosomes in OSCC. We found significant activation of intracellular MAPKs, especially p38 MAPK, in THP-1-derived macrophages treated by exosomes after 24 h (Additional file 1; Fig. 5a). Kinetic analysis of mRNA expression levels for TNFα, IL1β, and IL6 indicated the activation of macrophages occurred at the early phases after stimulation of exosomes (Fig. 5b). Accordingly, cell lysates of THP-1 derived macrophages were analyzed for intracellular signaling alterations after exosome treatment for 2 h and 6 h, and activation of p38 MAPK, Akt, and SAPK/JNK were observed (Fig. 5c). Immunoblotting assays further validated that activation of p38 MAPK, Akt, and SAPK/JNK signaling occurred at the early phase after the exosome stimulation (Fig. 5d).

### Exosome-mediated THBS1 transfer polarizes macrophages to an M1-like phenotype

**Fig. 6:**
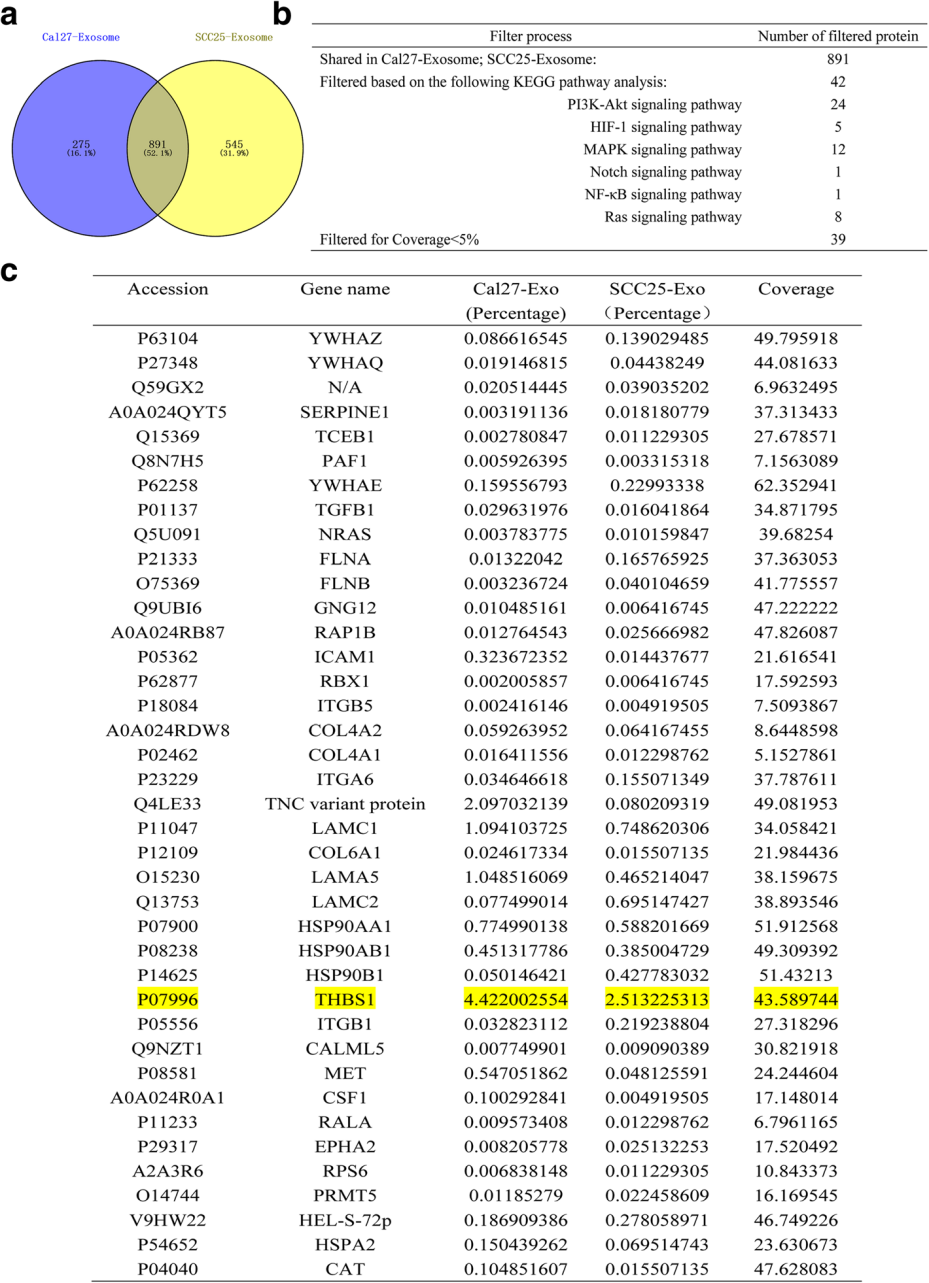
Quantitative proteomics analysis for exosomes extracted from CM of SCC25 and Cal27 cells. **a**. Venn diagram indicating 891 proteins shared between SCC25- and Cal27- exosomes. **b**. Proteins shared in SCC25- and Cal27- exosomes were filed based on KEGG pathway analysis by focusing on inflammatory related pathways, PI3K-Akt, HIF-1, MAPK, Notch, NF-κB, and Ras signaling pathways. Proteins with < 5% coverage were excluded. **c**. Among the filtered 39 proteins, functions were analyzed through UniProtKB. THBS1 was chosen for further analysis

Early-phase activation of p38 MAPK, Akt, and SAPK/JNK signaling suggested that the activation of macrophages might result from the direct action of effector proteins within the exosomes. Mass spectrometry based quantitative proteomics analysis of extracted exosomes revealed that 891 proteins were overlapped in both SCC25- and Cal27- derived exosomes (Fig. 6a). Proteins shared were then filtered by focusing on inflammatory-related pathways (PI3K-Akt, HIF-1, MAPKs, Notch, NF-κB, and Ras signaling pathways) according to KEGG analysis. Proteins with < 5% coverage were excluded. Eventually, 39 proteins remained and were subjected to further functional analysis through the UniProtKB database (Fig. 6b). We found that THBS1, accounting for the highest coverage among the 39 selected proteins, possesses positive regulation of macrophage activation from GO-biological process analysis [43].

**Fig. 7:**
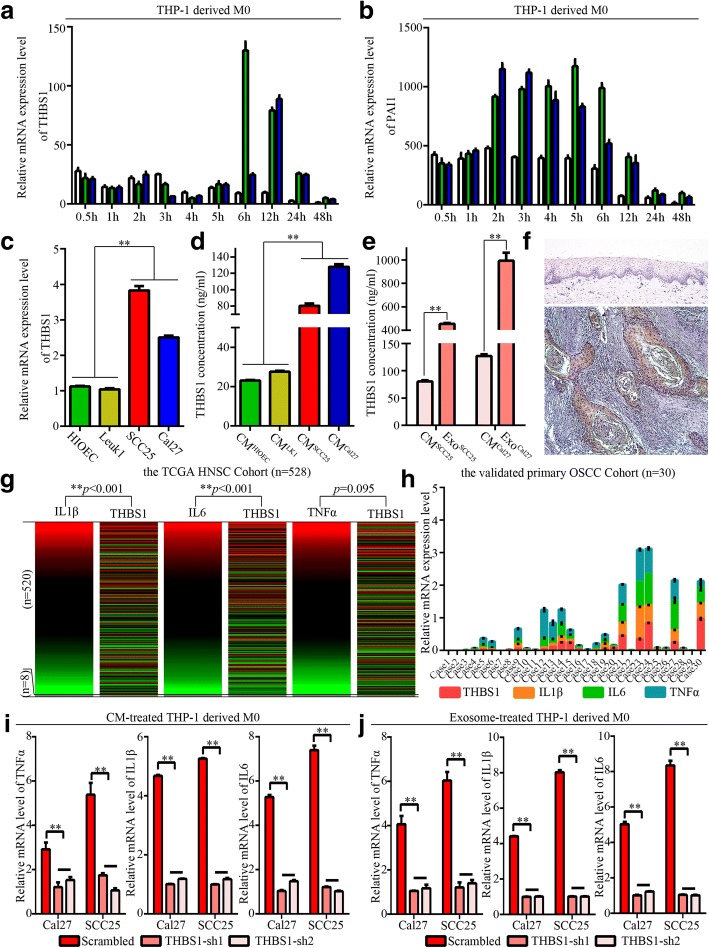
Exosomal transfer of THBS1 from OSCC activated macrophages into M1-like phenotype. **a-b**. THP-1-derived macrophages were treated with OSCC- exosomes for 0.5 h, 1 h, 2 h, 3 h, 4 h, 5 h, 6 h, 12 h, 24 h, and 48 h, respectively. Transcription levels of THBS1 and PAI1 were determined by qPCR. Data are representative of three independent experiments. **c**. Transcription levels of THBS1 in HIOEC, Leuk1, SCC25, and Cal27 cells. **d-e**. Expression levels of THBS1 were quantified in cellular culture supernatants and exosome suspensions by Platinum ELISA assays, **p < 0.01. **f**. Representative immunohistochemical staining demonstrating THBS1 was expressed by cancer cells in primary OSCC sample (100×). The upper part from normal oral epithelial tissue served as control; the lower part from primary OSCC tissue. **g**. Heat-map from the UCSC Xena Browser based on the primary TCGA HNSCC cohort depicted the gene expression relationship between TNFα, IL1β, or IL6 and THBS1, **p < 0.01. **h**. Expression pattern of THBS1, IL1β, IL6, and TNFα in a validated primary OSCC cohort (n = 30).**i-j**. Transcription levels for TNFα, IL1β, and IL6 in THP-1-derived macrophages educated by CM and exosome supernatants from THBS1-sh SCC25 and Cal27 cells for 24 h, respectively. Scrambled as control, **p < 0.01

THBS1 represents a potent pro-inflammatory signal for macrophages, and is also produced by macrophages [44, 45]. To determine that THBS1 was carried in exosomes, we detected the expression levels of THBS1 and its downstream molecule PAI1 in a time-dependent manner in macrophages activated by exosomes. We found that expression of THBS1 was increased 6 h after exosome treatment (Fig. 7a), while increased expression of PAI1 began at 2 h (Fig. 7b), suggesting that it was the THBS1 in the exosomes rather than the endogenous THBS1 in stimulated macrophages. Additionally, we observed higher levels of THBS1 in SCC5 and Cal27 cells than in HIOEC and Leuk1 cells (Fig. 7c, d). We observed significant accumulation of THBS1 in exosomes from SCC25 and Cal27 (Fig. 7e), suggesting that exosomes worked as effective cargo for THBS1 transfer from HNSCC cells. In primary OSCC tissues, THBS1 was primarily detected in cancer cells (Fig. 7f). We illustrated the expression relationship for THBS1 and M1-related genes in primary HNSCC TCGA cohort and primary OSCC validated cohort. We observed a significantly positive correlation between THBS1 and IL1β/IL6 expression in 520 primary HNSCC samples and 30 primary OSCC samples (Fig. 7g, h), indicating that higher THBS1 expression associated with increased M1 polarization status in primary OSCC. When knocked down expression of THBS1 in OSCC cells, the expression levels of TNFα, IL1β, and IL6 were significantly decreased for THP-1-derived macrophages stimulated by both indicated CM and exosome supernatants (Additional file 2; Fig. 7i, j).

### CMs from exosome-activated macrophages promote the migration of OSCC cells

**Fig. 8:**
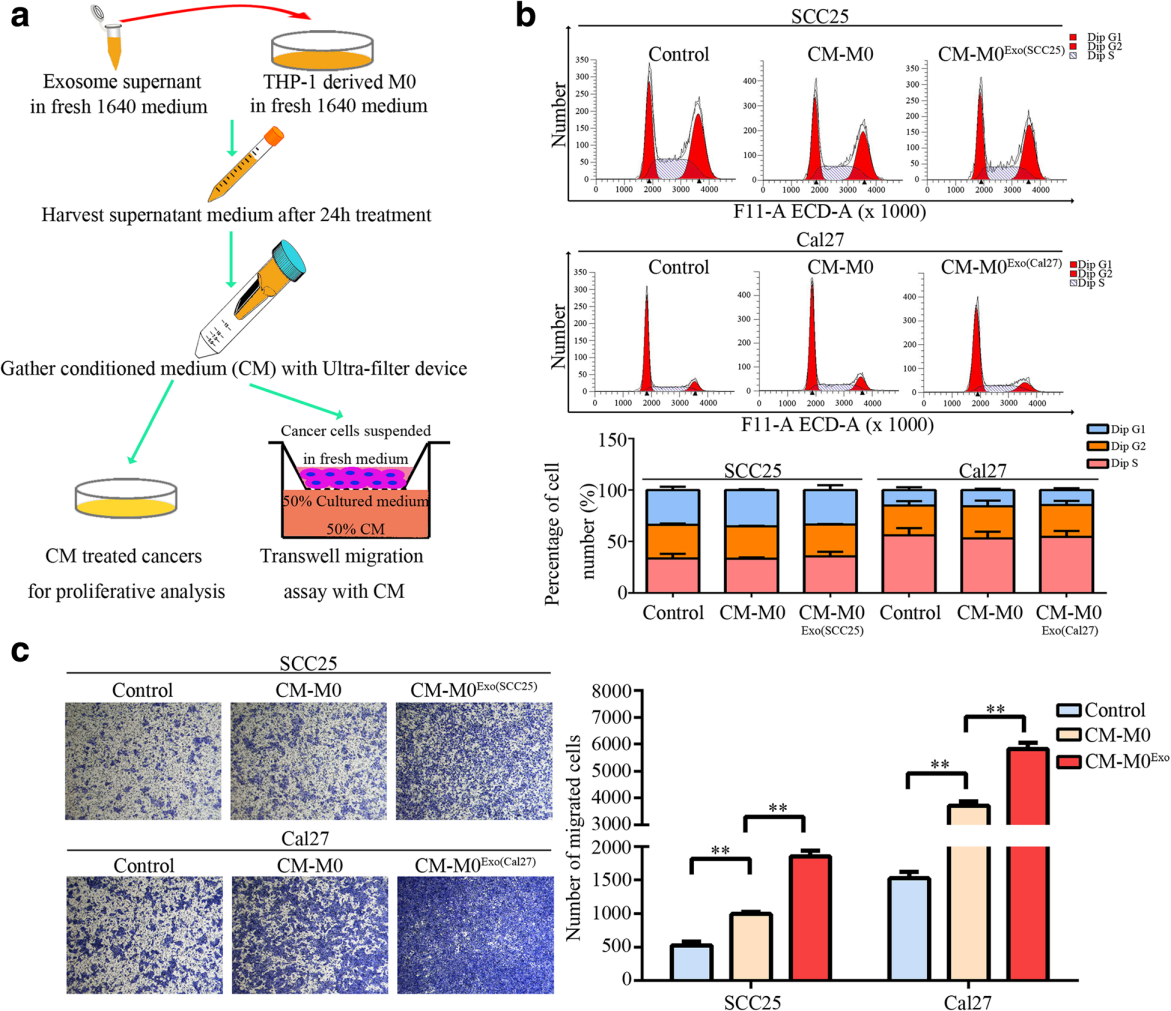
Effects of CM from exosome-activated macrophages on SCC25 and Cal27 cells. **a**. Scheme of experiments for CM from exosome-activated macrophages in the subsequent proliferation and migration analysis of OSCC cells. **b**. Cell cycle analysis by flow cytometry. Three independent experiments were performed, and a representative image was shown. No significant differences were observed. **c**. CM from exosome-treated macrophages increased motility of SCC25 and Cal27 cells. Three independent experiments were performed, and a representative image was shown, **p < 0.01

TAMs have been recognized to facilitate cancer-related malignancy in the tumor microenvironment [2, 7]. To study the effects of exosome-activated macrophages on the development of OSCC, CMs from exosome-activated macrophages were collected and treated to OSCC cells (Fig. 8a). Unde treatment, no significant responses were observed for the proliferative ability of SCC25 and Cal27 (Fig. 8b). However, the CMs significantly promoted the motility of OSCC cells (Fig. 8b). These data suggested that factors derived from macrophages exposed to exosomes played an important role in the malignant migration of OSCC.

## Discussion

**Fig. 9:**
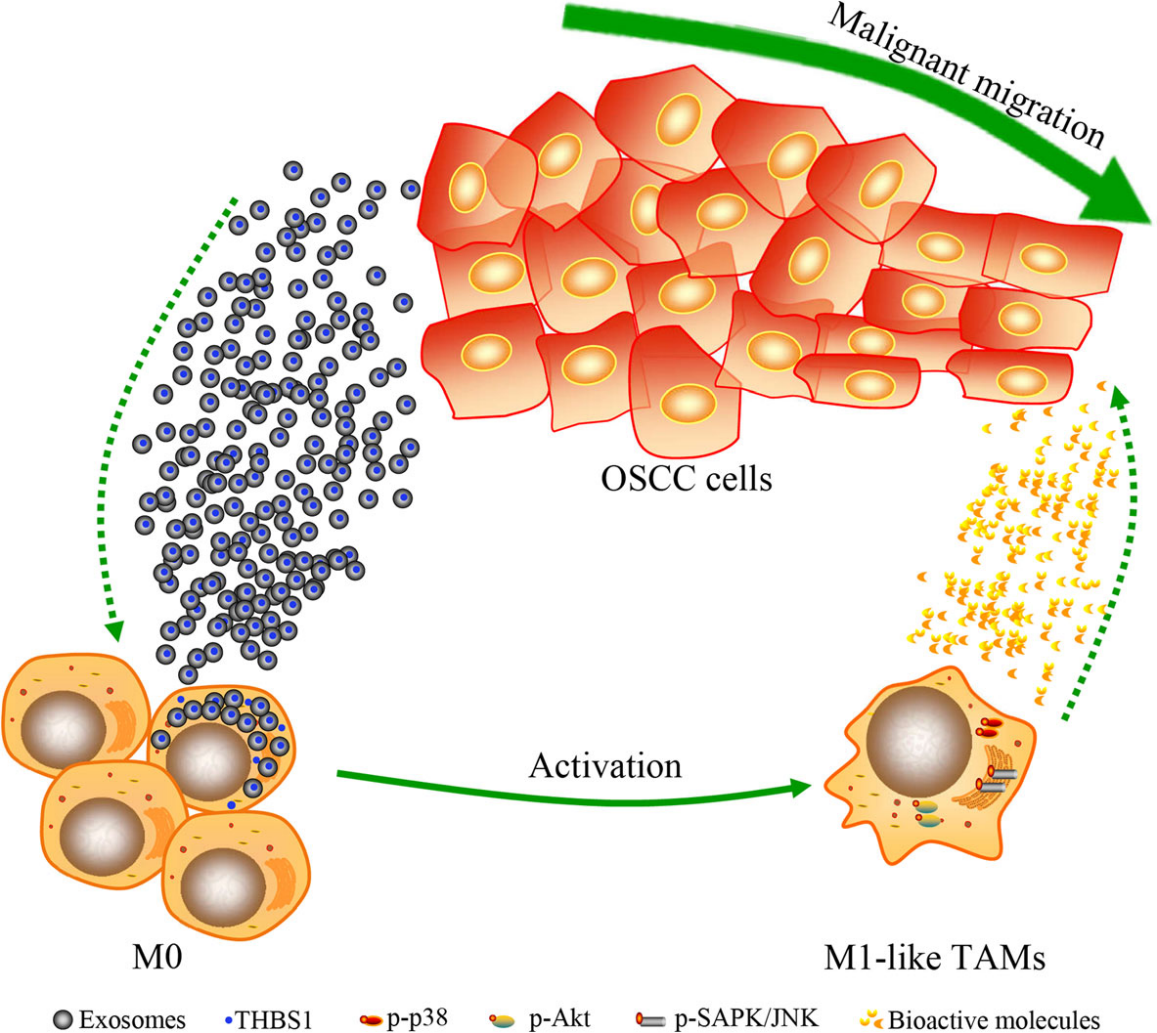
A schematic diagram for the novel paracrine loop identified between cancer cells and macrophages based on exosomes from OSCCs. Macrophages were activated into M1-like TAMs via uptake exosomal-THBS1 released from OSCC cells through p38, Akt, and SAPK/JNK signaling. Reciprocally, CM from the exosome-activated macrophages significantly promoted malignant migration of OSCC cells

Infiltration and polarization of macrophages is considered an essential event during carcinogenesis [12, 13], and the existence of paracrine loops between TAMs and cancer cells has been suggested [3, 15]. However, few studies have reported on TAM behaviors during epithelial cancer development. A representative OSCC-developing model was adopted to illustrate macrophage plasticity in this study. Here, we demonstrated that CM from OSCC cells activated macrophages to become M1-like phenotype, whereas no obvious functional changes were observed for macrophages treated with CM from precancerous or normal epithelial cells. In addition, M1-like TAMs were detected in primary OSCC samples. We showed that macrophage activation occurred by uptake of exosomes released from OSCC cells through p38, Akt, and SAPK/JNK signaling at the early phase. Further, we provided evidence that THBS1 derived from OSCC exosomes participated in the activation of macrophages to an M1-like phenotype. Reciprocally, CM from exosome-activated macrophages significantly promoted the malignant migration of OSCC cells. Therefore, we proposed a novel paracrine loop between cancer cells and macrophages based on exosomes from OSCCs (Fig. 9).

Herein, CM from normal epithelium, precancerous lesions, and oral cancers were harvested to educate macrophages in vitro, respectively. Unexpectedly, an obvious M1-like polarization status was observed only in CM-OSCC treated macrophages, suggesting that characterized activation of TAMs occurred after malignant transformation. Our results were in agreement with studies that have identified the presence and biological roles for a mixed population of macrophages with M1 and M2 phenotypes in cancers [17, 19, 20]. In malignant solid tumors, the education of TAMs is achieved by both direct contact through membrane molecules and paracrine loops between macrophages and cancer cells [3, 15, 46, 47]. We observed an early-phase M1-like phenotypic alteration under the treatment with CM-OSCC. In OSCC samples, M1-like TAMs accounted for about 31% of all macrophages, indicating the simultaneous existence of M2-like TAMs in OSCC. A previous study reported that M1-M2 transition of TAMS occurred during tumor progression, but the underlying signals involved in the M1/M2 switch are poorly understood [5]. Further studies are still required to determine the underlying mechanisms for the co-existence of M1 and M2-like TAMs in the tumor microenvironment of OSCC.

Previous studies have focused on soluble factors, i.e., cytokines, chemokines, and growth factors, produced by cancer cells. Until now, it has been suggested that exosomes derived from cancer cells have a wide range of biological functions [39, 48–50]. Exosomes are recognized as important signaling mediators, transferring lipids, proteins, mRNAs, microRNAs and lncRNAs to recipient cells, allowing the transfer of cancer-associated signaling molecules to surrounding cells, such as endothelial cells, immune cells, mesenchymal stromal cells, and etc. [39, 50–52]. Exosome-based communication in the microenvironments of cancer cells are some of the key events in cancer developing. In this study, we demonstrated that exosomes in CM from OSCC activated macrophages to M1-like phenotype.

We next applied intracellular signaling pathway analysis and found that exosomes from OSCC triggered macrophage polarization primarily through activation of p38, Akt, and SAPK/JNK signaling at an early-phase. The combined activation of Akt, p38 and JNK kinases participates in expression of pro-inflammatory mediators in macrophages [53]. Above all, these results provided evidences for the M1-like activation of macrophages in response to exosomes from OSCC. Furthermore, early-phase activation of macrophages indicated a direct and quick response to an effector protein carried by exosomes. THBS1 was identified via mass spectrometry based quantitative proteomics analysis. THBS1 is a multi-functional protein with potent pro-inflammatory and pro-migratory signaling effects on macrophages [43, 45, 54]. In addition, THBS1 has been identified as the most abundant protein secreted by OSCC and was reported to be significantly up-regulated in OSCC compared to normal epithelium [55]. Our study also identified higher mRNA expression and secreted levels of THBS1 in OSCC cells compared to pre-cancerous and normal epithelial cells. Additionally, bioinformatics analysis based on two cohorts and THBS1 knockdown assays indicated that THBS1 expression levels correlated well to the M1 activation in OSCC.

Previous studies have focused more on the roles of M2-like TAMs rather than M1-like TAMs in cancer pathology. We observed a centered distribution of M1-like macrophages in TAMs from OSCC samples. Therefore, we speculated that the biological effects of M1-like TAMs on cancer cells should be achieved through paracrine signaling. We discovered that exosome-activated macrophages could significantly promote the malignant migration of OSCC cells. M1 macrophages are always considered as key effector cells for the elimination of cancer cells either directly or indirectly through attraction and activation of NK and Th1 cells [7]. Hence, the M1-like TAMs activated by exosomes in OSCC may present therapeutic targets to motivate the tumoricidal potentials of these cells. In addition, this study highlighted the need for a more profound understanding of the roles and mechanisms of M1-like TAMs in OSCC.

## Conclusions

In conclusion, our findings demonstrated that exosomal transfer of THBS1 from oral cancer could polarize macrophages into M1-like TAMs. Targeted management of M1-like TAMs shows great potentials for the control of tumor cell migration in OSCCs.

## Additional files

Additional file 1: Heat-map indication of the densitometry values detected using the PathScan Immune Cell Signaling Antibody Array Kit. Colors illustrate fold changes (see color scale). Red: up-regulation; green: down-regulation. (DOCX 276 kb)

Additional file 2: Validation of THBS1 knockdown in SCC25 and Cal27 cells. A. Relative mRNA expression of THBS1 in SCC25 and Cal27 after THBS1 knockdown (Scrambled as control), as determined by quantitative real-time PCR. Data are represented as the mean ± SD of three independent experiments, **p < 0.01. B. Relative protein expression of THBS1 in SCC25 and Cal27 after knockdown of THBS1 (Scrambled as control), as determined by Western blotting. Data are represented as the mean ± SD of three independent experiments, **p < 0.01. C. Expression level of THBS1 in CM of SCC25 and Cal27 after THBS1 knockdown (Scrambled as control), as determined by ELISA assays, **p < 0.01. D. Expression level of THBS1 in exosome supernatants of SCC25 and Cal27 cells after THBS1 knockdown (Scrambled as control), as determined by ELISA assays, **p < 0.01. (DOCX 199 kb)

Additional file 3: Negative control for tracing exosome uptake by macrophages. Cultured macrophages were fixed, permeabilized, and stained with Acti-stain™ 488-Phalloidin and DAPI. Then, these macrophages were examined under confocal microscope. No red signals were captured in macrophages with an excitation at 460 nm without the incubation of labelled exosomes. (DOCX 240 kb)

Additional file 4: Control images for the Chromogenic double staining with CD80 (pink)/CD68 (brown) in primary OSCC samples. Sections stained for hematoxylin were used as negative control. Sections stained with CD68 were used as single-positive control. (DOCX 557 kb)

## Abbreviations

ATCC: American Type Culture Collection
CM: Conditioned media
ELISA: Enzyme-linked Immunosorbent Assay
HIOEC: Human immortal oral epithelial cell line cells
HNSCC: Head and neck squamous cell carcinoma
IHC: Immunohistochemistry
Leuk1: Oral leukoplakia cell line cells
MACS: Magnetic activated cell sorting
MS: Mass spectrometry
NTA: Nanoparticle tracking analysis
OSCC: Oral squamous cell carcinoma
PBMCs: Peripheral blood mononuclear cells
PMA: Phorbol-12-myristate-13-acetate
qRT-PCR: Quantitative real-time PCR
TAMs: Tumor-associated macrophages

## References

[1] Chen DS, Mellman I (2017). Elements of cancer immunity and the cancer-immune set point. Nat..

[2] Mantovani A, Allavena P, Sica A, Balkwill F (2008). Cancer-related inflammation. Nat.

[3] Wyckoff J, Wang W, Lin EY, Wang Y, Pixley F, Stanley ER (2004). A paracrine loop between tumor cells and macrophages is required for tumor cell migration in mammary tumors. Cancer Res.

[4] Solinas G, Schiarea S, Liguori M, Fabbri M, Pesce S, Zammataro L (2010). Tumor-conditioned macrophages secrete migration-stimulating factor: a new marker for M2-polarization, influencing tumor cell motility. J Immunol.

[5] Schmieder A, Michel J, Schonhaar K, Goerdt S, Schledzewski K (2012). Differentiation and gene expression profile of tumor-associated macrophages. Semin Cancer Biol.

[6] Sousa S, Brion R, Lintunen M, Kronqvist P, Sandholm J, Monkkonen J (2015). Human breast cancer cells educate macrophages toward the M2 activation status. Breast Cancer Res.

[7] Heusinkveld M, van der Burg SH (2011). Identification and manipulation of tumor associated macrophages in human cancers. J Transl Med.

[8] Schultze JL, Schmidt SV (2015). Molecular features of macrophage activation. Semin Immunol.

[9] Murray PJ, Allen JE, Biswas SK, Fisher EA, Gilroy DW, Goerdt S (2014). Macrophage activation and polarization: nomenclature and experimental guidelines. Immunity.

[10] Italiani P, Boraschi D (2014). From monocytes to M1/M2 macrophages: phenotypical vs. functional differentiation. Front Immuno.

[11] Xu H, Zhu J, Smith S, Foldi J, Zhao B, Chung AY (2012). Notch-RBP-J signaling regulates the transcription factor IRF8 to promote inflammatory macrophage polarization. Nat Immunol.

[12] Karnevi E, Andersson R, Rosendahl AH (2014). Tumour-educated macrophages display a mixed polarisation and enhance pancreatic cancer cell invasion. Immunol Cell Biol.

[13] Miyashita T, Tajima H, Shah FA, Oshima M, Makino I, Nakagawara H (2014). Impact of inflammation-metaplasia-adenocarcinoma sequence and inflammatory microenvironment in esophageal carcinogenesis using surgical rat models. Ann Surg Oncol.

[14] Philip PA (2016). Targeting macrophages to treat pancreatic cancer. Lancet Oncol.

[15] Zhao JL, Huang F, He F, Gao CC, Liang SQ, Ma PF (2016). Forced activation of notch in macrophages represses tumor growth by upregulating miR-125a and disabling tumor-associated macrophages. Cancer Res.

[16] Zhang Q, Cai DJ, Li B (2015). Ovarian cancer stem-like cells elicit the polarization of M2 macrophages. Mol Med Rep.

[17] Wang H, Wang X, Li X, Fan Y, Li G, Guo C (2014). CD68(+)HLA-DR(+) M1-like macrophages promote motility of HCC cells via NF-kappaB/FAK pathway. Cancer Lett.

[18] Estko M, Baumgartner S, Urech K, Kunz M, Regueiro U, Heusser P (2015). Tumour cell derived effects on monocyte/macrophage polarization and function and modulatory potential of Viscum album lipophilic extract in vitro. BMC Complem Altern M.

[19] Wu TH, Li YY, Wu TL, Chang JW, Chou WC, Hsieh LL (2014). Culture supernatants of different colon cancer cell lines induce specific phenotype switching and functional alteration of THP-1 cells. Cell Immunol.

[20] Helm O, Held-Feindt J, Grage-Griebenow E, Reiling N, Ungefroren H, Vogel I (2014). Tumor-associated macrophages exhibit pro- and anti-inflammatory properties by which they impact on pancreatic tumorigenesis. Int J Cancer.

[21] Siegel RL, Miller KD, Jemal A (2016). Cancer statistics, 2016. CA-Cancer J Clin.

[22] Saintigny P, Zhang L, Fan YH, El-Naggar AK, Papadimitrakopoulou VA, Feng L (2011). Gene expression profiling predicts the development of oral cancer. Cancer Prev Res.

[23] Go A, Ryu YK, Lee JW, Moon EY (2013). Cell motility is decreased in macrophages activated by cancer cell-conditioned medium. Biomol Ther.

[24] Cao W, Younis RH, Li J, Chen H, Xia R, Mao L (2011). EZH2 promotes malignant phenotypes and is a predictor of oral cancer development in patients with oral leukoplakia. Cancer Prev Res.

[25] Cao W, Zhang ZY, Xu Q, Sun Q, Yan M, Zhang J (2010). Epigenetic silencing of MAL, a putative tumor suppressor gene, can contribute to human epithelium cell carcinoma. Mol Cancer.

[26] Wu X, Cao W, Wang X, Zhang J, Lv Z, Qin X (2013). TGM3, a candidate tumor suppressor gene, contributes to human head and neck cancer. Mol Cancer.

[27] Richter E, Ventz K, Harms M, Mostertz J, Hochgrafe F (2016). Induction of macrophage function in human THP-1 cells is associated with rewiring of MAPK signaling and activation of MAP3K7 (TAK1) protein kinase. Front Cell Dev Biol.

[28] Stewart DA, Yang Y, Makowski L, Troester MA (2012). Basal-like breast cancer cells induce phenotypic and genomic changes in macrophages. Mol Cancer Res.

[29] Qin Z (2012). The use of THP-1 cells as a model for mimicking the function and regulation of monocytes and macrophages in the vasculature. Atherosclerosis.

[30] Fang S, Huang Y, Zhong S, Zhang Y, Liu X, Wang Y (2016). IL-17A promotes RANTES expression, but not IL-16, in orbital fibroblasts via CD40-CD40L combination in thyroid-associated Ophthalmopathy. Invest Ophth Vis Sci.

[31] Pello OM, De Pizzol M, Mirolo M, Soucek L, Zammataro L, Amabile A (2012). Role of c-MYC in alternative activation of human macrophages and tumor-associated macrophage biology. Blood.

[32] Vogel DY, Glim JE, Stavenuiter AW, Breur M, Heijnen P, Amor S (2014). Human macrophage polarization in vitro: maturation and activation methods compared. Immunobiology.

[33] Lobb RJ, Becker M, Wen SW, Wong CS, Wiegmans AP, Leimgruber A (2015). Optimized exosome isolation protocol for cell culture supernatant and human plasma. J Extracell Vesicles.

[34] Harp D, Driss A, Mehrabi S, Chowdhury I, Xu W, Liu D (2016). Exosomes derived from endometriotic stromal cells have enhanced angiogenic effects in vitro. Cell Tissue Res.

[35] Anand S, Samuel M, Ang CS, Keerthikumar S, Mathivanan S (2017). Label-based and label-free strategies for protein quantitation. Methods Mol Biol.

[36] Goldman M, Craft B, Swatloski T, Cline M, Morozova O, Diekhans M (2015). The UCSC Cancer genomics browser: update 2015. Nucleic Acids Res.

[37] Zhu J, Sanborn JZ, Benz S, Szeto C, Hsu F, Kuhn RM (2009). The UCSC Cancer genomics browser. Nat Methods.

[38] Baglio SR, Rooijers K, Koppers-Lalic D, Verweij FJ, Perez Lanzon M, Zini N (2015). Human bone marrow- and adipose-mesenchymal stem cells secrete exosomes enriched in distinctive miRNA and tRNA species. Stem Cell Res Ther.

[39] Lin LY, Du LM, Cao K, Huang Y, Yu PF, Zhang LY (2016). Tumour cell-derived exosomes endow mesenchymal stromal cells with tumour-promotion capabilities. Oncogene.

[40] de Couto G, Gallet R, Cambier L, Jaghatspanyan E, Makkar N, Dawkins JF (2017). Exosomal microRNA transfer into macrophages mediates cellular Postconditioning. Circulation.

[41] Srinivasan S, Vannberg FO, Dixon JB (2016). Lymphatic transport of exosomes as a rapid route of information dissemination to the lymph node. Sci Rep.

[42] Cui YL, Li HK, Zhou HY, Zhang T, Li Q (2013). Correlations of tumor-associated macrophage subtypes with liver metastases of colorectal cancer. Asian Pac J Cancer P.

[43] Martin-Manso G, Galli S, Ridnour LA, Tsokos M, Wink DA, Roberts DD (2008). Thrombospondin 1 promotes tumor macrophage recruitment and enhances tumor cell cytotoxicity of differentiated U937 cells. Cancer Res.

[44] Yamauchi Y, Kuroki M, Imakiire T, Abe H, Uchida H, Beppu R (2002). Thrombospondin-1 differentially regulates release of IL-6 and IL-10 by human monocytic cell line U937. Biochem Bioph Res Co.

[45] Urao N, Mirza RE, Heydemann A, Garcia J, Koh TJ (2016). Thrombospondin-1 levels correlate with macrophage activity and disease progression in dysferlin deficient mice. Neuromuscul Disord.

[46] Singla RD, Wang J, Singla DK (2014). Regulation of notch 1 signaling in THP-1 cells enhances M2 macrophage differentiation. Am J Physiol-Heart C.

[47] Zhang Y, Sime W, Juhas M, Sjolander A (2013). Crosstalk between colon cancer cells and macrophages via inflammatory mediators and CD47 promotes tumour cell migration. Eur J Cancer.

[48] Kourembanas S (2015). Exosomes: vehicles of intercellular signaling, biomarkers, and vectors of cell therapy. Annu Rev Physiol.

[49] Isola AL, Chen S (2016). Exosomes: the link between GPCR activation and metastatic potential?. Front Genet.

[50] Berchem G, Noman MZ, Bosseler M, Paggetti J, Baconnais S, Le Cam E (2015). Hypoxic tumor-derived microvesicles negatively regulate NK cell function by a mechanism involving TGF-beta and miR23a transfer. Oncoimmunology.

[51] Hannafon BN, Carpenter KJ, Berry WL, Janknecht R, Dooley WC, Ding WQ (2015). Exosome-mediated microRNA signaling from breast cancer cells is altered by the anti-angiogenesis agent docosahexaenoic acid (DHA). Mol Cancer.

[52] He M, Qin H, Poon TC, Sze SC, Ding X, Co NN (2015). Hepatocellular carcinoma-derived exosomes promote motility of immortalized hepatocyte through transfer of oncogenic proteins and RNAs. Carcinogenesis.

[53] Ivashkiv LB (2011). Inflammatory signaling in macrophages: transitions from acute to tolerant and alternative activation states. Eur J Immunol.

[54] Liu Z, Morgan S, Ren J, Wang Q, Annis DS, Mosher DF (2015). Thrombospondin-1 (TSP1) contributes to the development of vascular inflammation by regulating monocytic cell motility in mouse models of abdominal aortic aneurysm. Circ Res.

[55] Pal SK, Nguyen CT, Morita KI, Miki Y, Kayamori K, Yamaguchi A (2016). THBS1 is induced by TGFB1 in the cancer stroma and promotes invasion of oral squamous cell carcinoma. J Oral Pathol Med.

