# Supplementary material for: M1-like tumor-associated macrophages activated by exosome-transferred THBS1 promote malignant migration in oral squamous cell carcinoma
Source: J Exp Clin Cancer Res. 2018 Jul 9;37:143. doi: 10.1186/s13046-018-0815-2 (PMC6038304; doi:10.1186/s13046-018-0815-2)
Supplement: Supplementary file 1 — Heat-map indication of the densitometry values detected using the PathScan Immune Cell Signaling Antibody Array Kit. Colors illustrate fold changes (see color scale). Red: up-regulation; green: down-regulation. (DOCX 276 kb) [file 13046_2018_815_MOESM1_ESM.docx]

**Additional file 1**

**
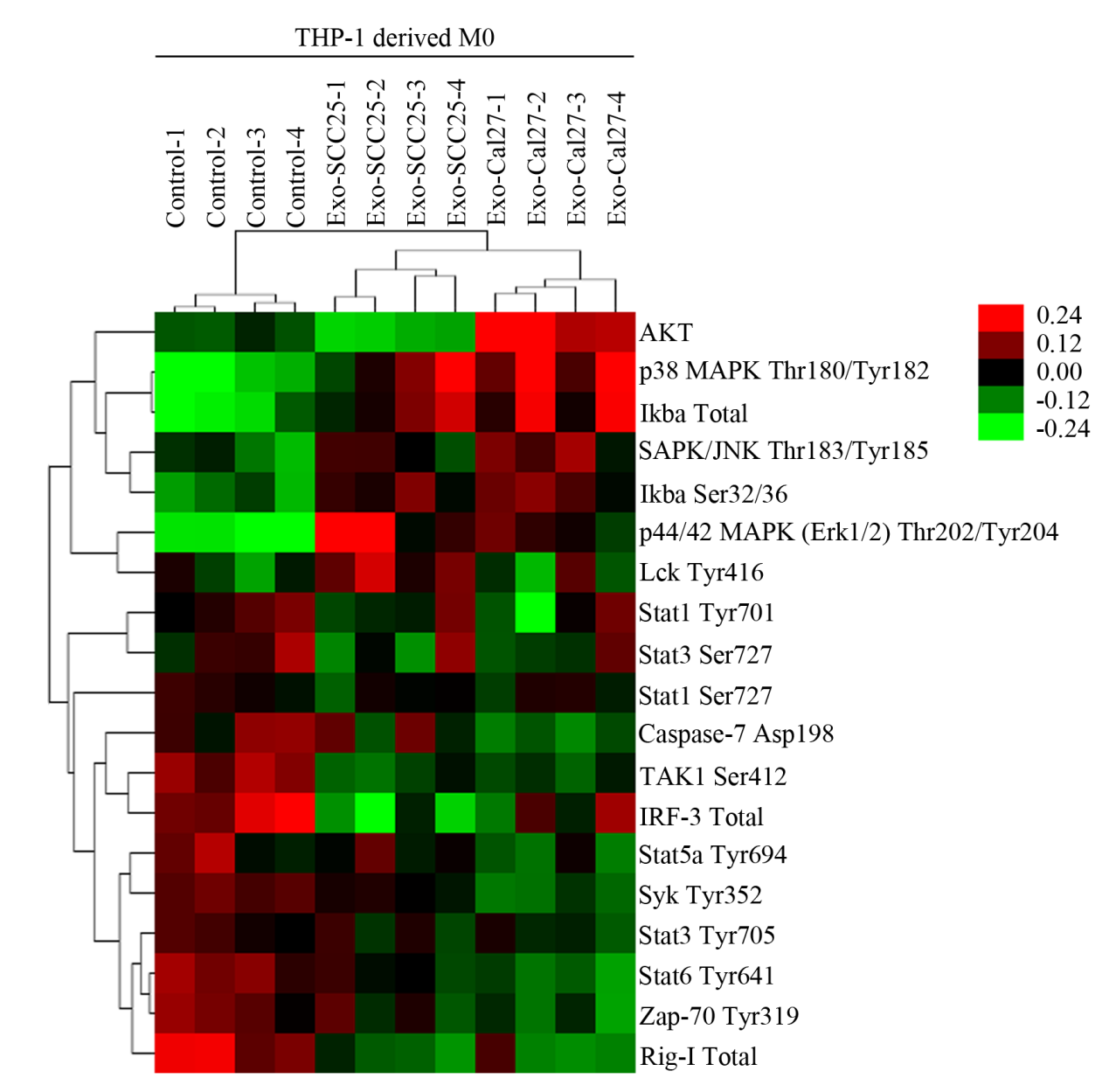
**

Additional file 3: Heat-map indication of the densitometry values detected using the PathScan Immune Cell Signaling Antibody Array Kit. Colors illustrate fold changes (see color scale). Red: up-regulation; green: down-regulation.
