# Supplementary material for: M1-like tumor-associated macrophages activated by exosome-transferred THBS1 promote malignant migration in oral squamous cell carcinoma
Source: J Exp Clin Cancer Res. 2018 Jul 9;37:143. doi: 10.1186/s13046-018-0815-2 (PMC6038304; doi:10.1186/s13046-018-0815-2)
Supplement: Supplementary file 2 — Validation of THBS1 knockdown in SCC25 and Cal27 cells. A. Relative mRNA expression of THBS1 in SCC25 and Cal27 after THBS1 knockdown (Scrambled as control), as determined by quantitative real-time PCR. Data are represented as the mean ± SD of three independent experiments, **p < 0.01. B. Relative protein expression of THBS1 in SCC25 and Cal27 after knockdown of THBS1 (Scrambled as control), as determined by Western blotting. Data are represented as the mean ± SD of three independent experiments, **p < 0.01. C. Expression level of THBS1 in CM of SCC25 and Cal27 after THBS1 knockdown (Scrambled as control), as determined by ELISA assays, **p < 0.01. D. Expression level of THBS1 in exosome supernatants of SCC25 and Cal27 cells after THBS1 knockdown (Scrambled as control), as determined by ELISA assays, **p < 0.01. (DOCX 199 kb) [file 13046_2018_815_MOESM2_ESM.docx]

**Additional file 2**

**
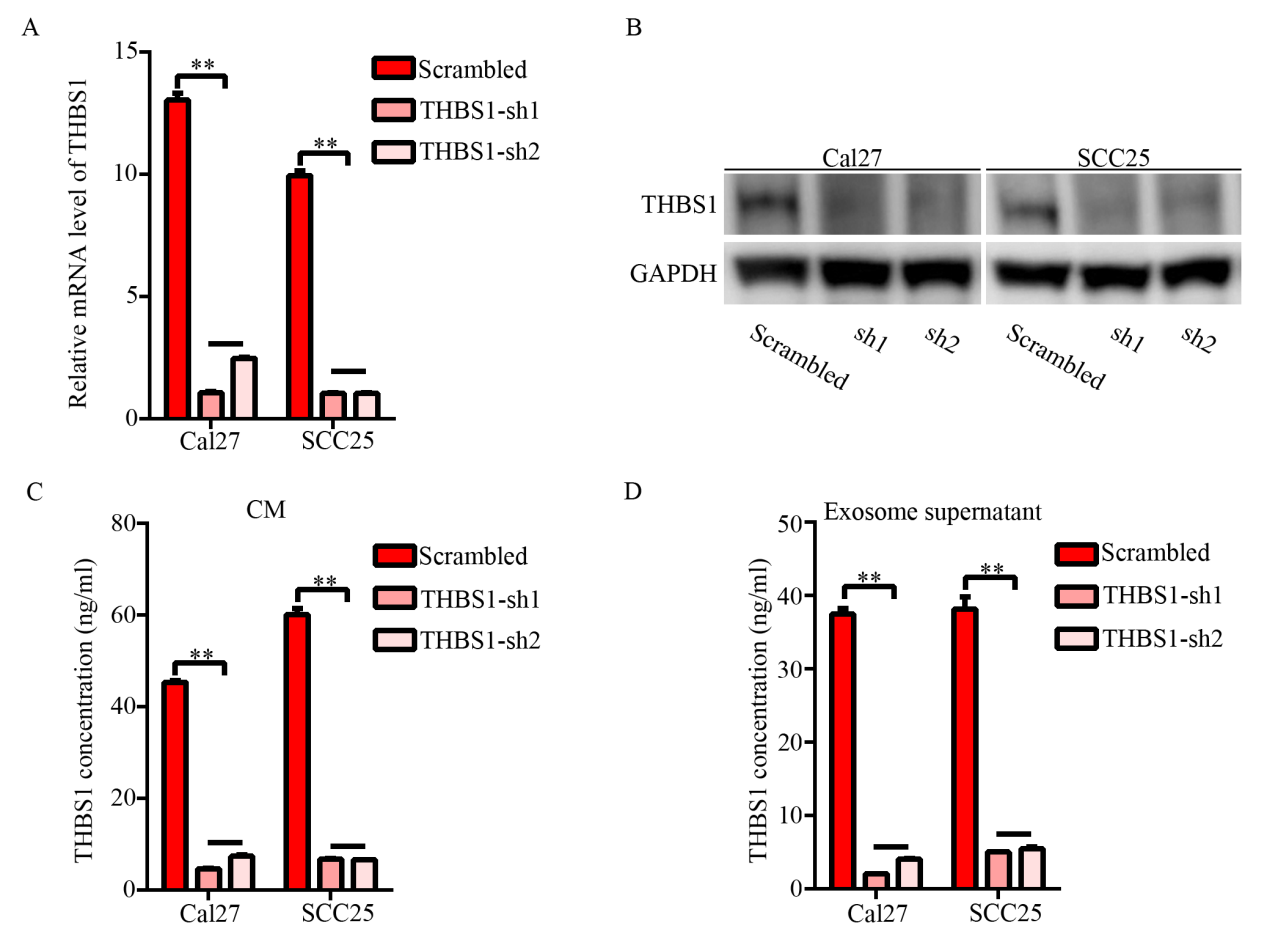
**

Additional file 4: Validation of THBS1 knockdown in SCC25 and Cal27 cells. A. Relative mRNA expression of THBS1 in SCC25 and Cal27 after THBS1 knockdown (Scrambled as control), as determined by quantitative real-time PCR. Data are represented as the mean ± SD of three independent experiments, ***p*<0.01. B. Relative protein expression of THBS1 in SCC25 and Cal27 after knockdown of THBS1 (Scrambled as control), as determined by Western blotting. Data are represented as the mean ± SD of three independent experiments, ***p*<0.01. C. Expression level of THBS1 in CM of SCC25 and Cal27 after THBS1 knockdown (Scrambled as control), as determined by ELISA assays, ***p*<0.01. D. Expression level of THBS1 in exosome supernatants of SCC25 and Cal27 cells after THBS1 knockdown (Scrambled as control), as determined by ELISA assays, ***p*<0.01.
