# Supplementary material for: M1-like tumor-associated macrophages activated by exosome-transferred THBS1 promote malignant migration in oral squamous cell carcinoma
Source: J Exp Clin Cancer Res. 2018 Jul 9;37:143. doi: 10.1186/s13046-018-0815-2 (PMC6038304; doi:10.1186/s13046-018-0815-2)
Supplement: Supplementary file 3 — Negative control for tracing exosome uptake by macrophages. Cultured macrophages were fixed, permeabilized, and stained with Acti-stain™ 488-Phalloidin and DAPI. Then, these macrophages were examined under confocal microscope. No red signals were captured in macrophages with an excitation at 460 nm without the incubation of labelled exosomes. (DOCX 240 kb) [file 13046_2018_815_MOESM3_ESM.docx]

**Additional file 3**

**
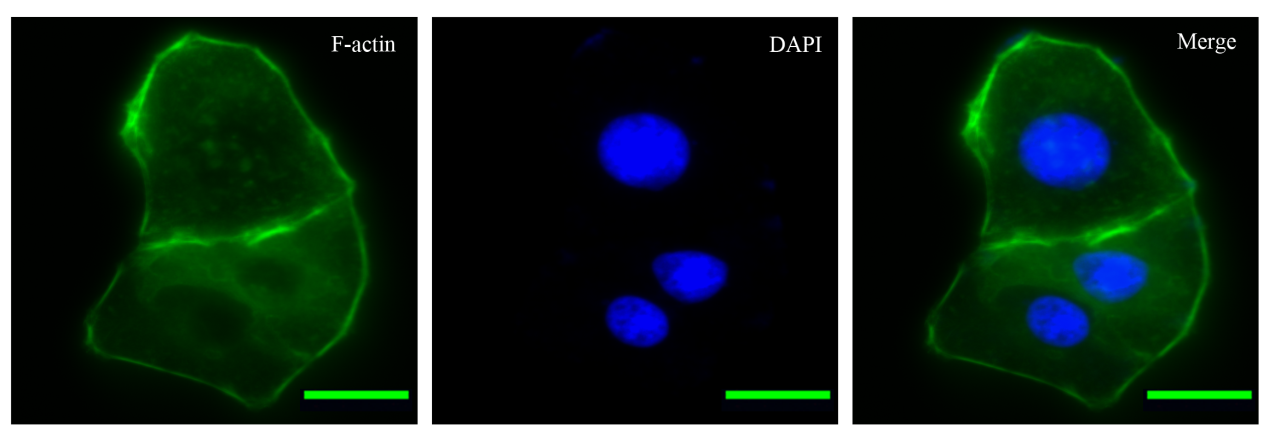
**

Additional file 1: Negative control for tracing exosome uptake by macrophages. Cultured macrophages were fixed, permeabilized, and stained with Acti-stain^TM^ 488-Phalloidin and DAPI. Then, these macrophages were examined under confocal microscope. No red signals were captured in macrophages with an excitation at 460nm without the incubation of labelled exosomes.
