# Supplementary material for: M1-like tumor-associated macrophages activated by exosome-transferred THBS1 promote malignant migration in oral squamous cell carcinoma
Source: J Exp Clin Cancer Res. 2018 Jul 9;37:143. doi: 10.1186/s13046-018-0815-2 (PMC6038304; doi:10.1186/s13046-018-0815-2)
Supplement: Supplementary file 4 — Control images for the Chromogenic double staining with CD80 (pink)/CD68 (brown) in primary OSCC samples. Sections stained for hematoxylin were used as negative control. Sections stained with CD68 were used as single-positive control. (DOCX 557 kb) [file 13046_2018_815_MOESM4_ESM.docx]

**Additional file 4**

**
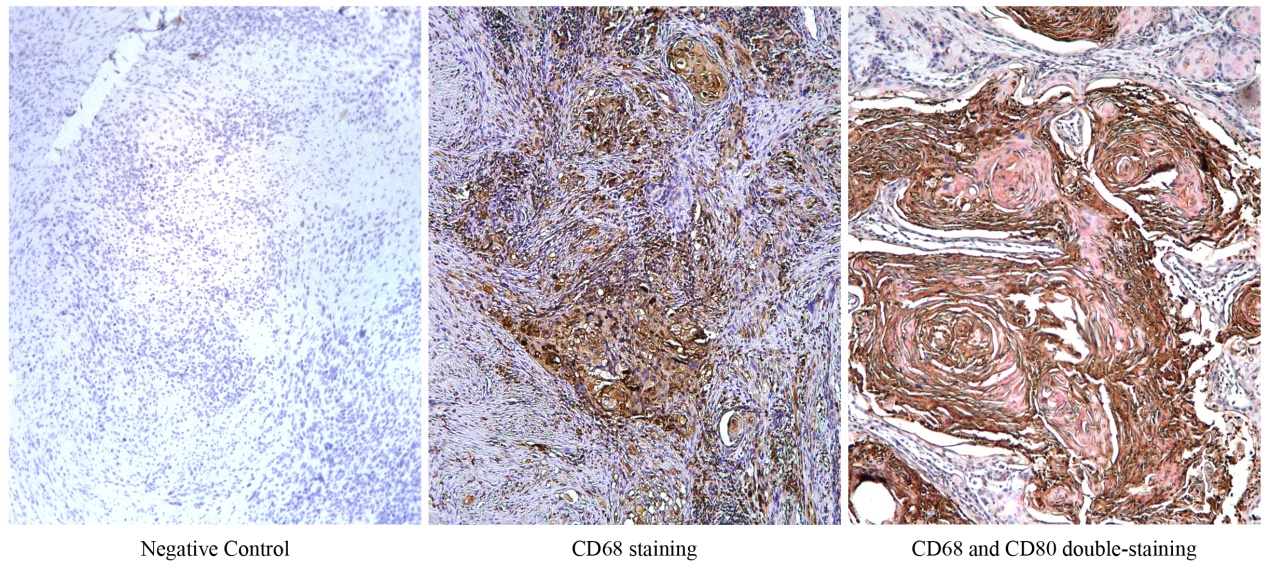
**

Additional file 2: Control images for the Chromogenic double staining with CD80 (pink)/CD68 (brown) in primary OSCC samples. Sections stained for hematoxylin were used as negative control. Sections stained with CD68 were used as single-positive control.
